# Bronchoscope-guided percutaneous dilatational tracheostomy performed by an experienced intensivist: a 26-month experience at a tertiary care center in United Arab Emirates

**DOI:** 10.1186/cc10749

**Published:** 2012-03-20

**Authors:** M Rahman, R Ammar, D Abdullah, F Chedid, S Abuhasna

**Affiliations:** 1 Tawam Hospital, Al Ain, United Arab Emirates

## Introduction

Bedside percutaneous dilatational tracheostomy (PDT) is a safe procedure with an acute complication rate of 10 to 15%. Our hypothesis was that having an experienced person performing or supervising the procedure results in extremely low complications with PDT. We formed a tracheostomy team which always included at least a consultant or specialist experienced (at least 25 procedures) in performing the procedure.

## Methods

A retrospective chart review of all patients who had PDT in a multidisciplinary adult medical surgical ICU during November 2008 to December 2010. The patients' demographics, indications for intubation and PDT, early and late complications, date weaned off the ventilator, date of decannulation, discharge from ICU and hospital, and outcome of these patients in the hospital were noted.

## Results

Out of a total of 2,364 admission 57 patients underwent PDT, all with bronchoscopic guidance by an intensivist experienced in PDT (>25 procedures); there were 45 (78.9%) males and 12 (21%) females with the median age of 42 (range 18 to 90) years. The most common admission diagnosis was cardiac arrest *n *= 14 (24%) followed by severe head injury *n *= 13(23%) and cerebrovascular accident *n *= 8 (14%). The commonest indication for tracheostomy was airway protection *n *= 40 (73%) followed by prolonged mechanical ventilation *n *= 25 (45%). The median duration of intubation before PDT was 11 days (IQ 8 to 18). The median time elapsed between tracheostomy and weaning of ventilator was 1 day (IQ 1 to 3). However, the median time to decannulation was 37 day (IQ 10 to 136). Acute complication of paratracheal insertion occurred in *n *= 1 (1.8%) patient. No deaths were reported related to the procedure. However, *n *= 13 (22.8%) patients died during the hospital stay. No procedure was converted to surgical tracheostomy. The median duration between tracheostomy and discharge from ICU was 12 days (IQ 5 to 21). Chronic complication of subglottic stenosis occurred in *n *= 1 (1.8%) patient.

## Conclusion

PDT is an extremely safe procedure when performed by an experienced intensivist under bronchoscopic guidance. Our low complication rate is due to careful screening and selection of patients and being performed or supervised by an experienced intensivist under direct vision.

